# Supplementary material for: Shared Treg dysregulation underlies psoriasis vulgaris and atopic dermatitis through the PHF19-PRC2 epigenetic axis
Source: Front Immunol. 2026 Aug 20;17:1932788. doi: 10.3389/fimmu.2026.1932788 (PMC13538097; doi:10.3389/fimmu.2026.1932788)
Supplement: Supplementary file 3 [file DataSheet3.zip › Additional files/Additional file 1/16.ETFB.scatter.pdf]

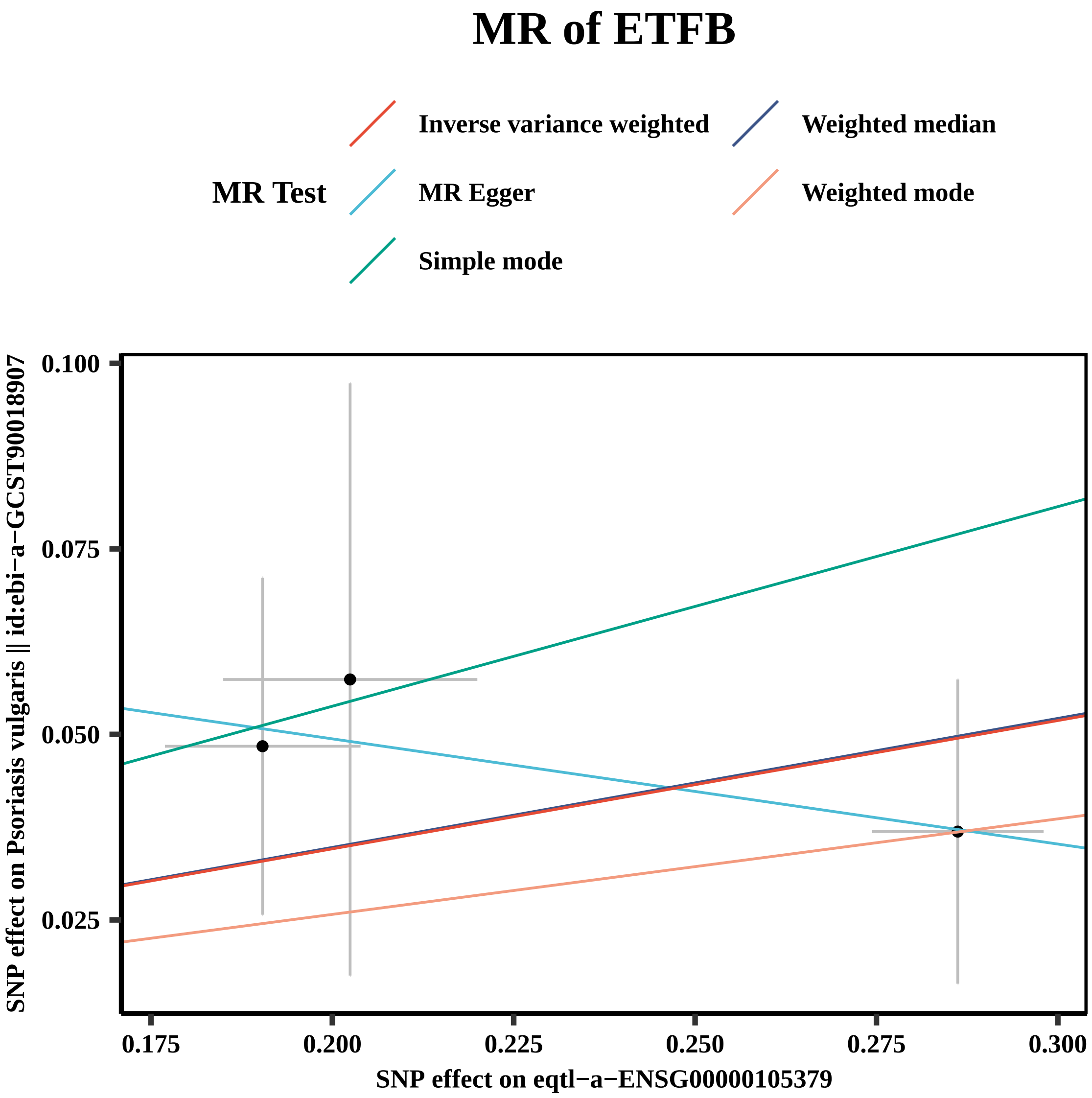
MR of ETFB
MR Test
Inverse variance weighted
MR Egger
Simple mode
Weighted median
Weighted mode
0.100
0.075
0.050
0.025
0.175
0.200
0.225
0.250
0.275
0.300
SNP effect on Psoriasis vulgaris || id:ebi−a−GCST90018907
SNP effect on eqtl−a−ENSG00000105379
